# A Newly Isolated Carboxymethyl-Glucan (CM-G) Restores Depressed Baroreflex Sensitivity in Renovascular Hypertensive Rats

**DOI:** 10.3389/fphys.2018.00607

**Published:** 2018-05-23

**Authors:** Alynne Carvalho-Galvão, Danilo D. A. Gadelha, José L. de Brito Alves, Barkat A. Khan, Raul J. H. Castro-Gomez, Josiane C. Cruz, Marciane Magnani, Valdir A. Braga

**Affiliations:** ^1^Department of Biotechnology, Center of Biotechnology, Federal University of Paraíba, João Pessoa, Brazil; ^2^Department of Nutrition, Health Sciences Center, Federal University of Paraíba, João Pessoa, Brazil; ^3^Faculty of Pharmacy, Gomal University, Dera Ismail Khan, Pakistan; ^4^Department of Foods Engineering, Technology Center, Federal University of Paraíba, João Pessoa, Brazil

**Keywords:** hypertension, sympathetic overactivity, baroreflex sensitivity, carboxymethyl-glucan

## Abstract

This study was designed to investigate the effects of a newly synthesized carboxymethyl-glucan (CM-G) on blood pressure (BP), baroreflex sensitivity (BRS) and sympathetic vascular modulation in renovascular hypertensive rats. Male Wistar rats were divided into four groups: Sham (*n* = 10); 2K1C (subjected to renal artery clipping to induce renovascular hypertension, *n* = 10); Sham + CM-G (treated with CM-G, *n* = 7) and 2K1C + CM-G (treated with CM-G, *n* = 7). The daily treatment with CM-G (40 mg/kg) was performed for 2 weeks. Blood pressure, heart rate (HR), systolic BP variability, baroreflex sensitivity (BRS) and sympathetic vascular tone were evaluated. After six weeks of renal artery clipping, 2K1C rats exhibited arterial hypertension (171 ± 11 vs. 118 ± 4 mmHg, p < 0.05), impaired BRS (-1.30 ± 0.10 vs. -2.59 ± 0.17 bpm.mmHg-1, p < 0.05) and enhanced sympathetic activity as shown by the hexamethonium test (-60 ± 5 vs. -33 ± 2 ΔmmHg, p < 0.05) when compared to sham rats. Oral administration of CM-G in renovascular hypertensive rats reduced hypertension (126 ± 4 vs. 171 ± 11 mmHg, p < 0.05) and improved the BRS (-2.03 ± 0.16 vs. -1.30 ± 0.10 bpm.mmHg^-1^, p < 0.05) in 2K1C rats when compared to placebo. Those effects seem to be caused by a reduction in sympathetic activity. The present study revealed for the first time that CM-G treatment reduces arterial hypertension and restores arterial baroreflex sensitivity via a reduction in the sympathetic tone in conscious renovascular hypertensive rats.

## Introduction

Baroreflex is essential for the short-term control of blood pressure (BP) and modulation of sympathetic activity (Malpas et al., 1997; Grassi et al., 1998; Julien, 2008). Convincing evidence from research with both animals and humans have reported a relationship between decreased arterial baroreflex sensitivity, sympathetic overactivity and arterial hypertension (Gao et al., 2002; Nagai et al., 2003; Salgado et al., 2007).

Our laboratory and other research groups have demonstrated that augmented oxidative stress, characterized by downregulation of the antioxidant capacity and/or increased pro-oxidant factors, depresses baroreflex sensitivity, promotes endothelial dysfunction, augments sympathetic activity and may contribute to the development and maintenance of arterial hypertension (Guimaraes et al., 2012; de Queiroz et al., 2015; Cavalcanti et al., 2016). The role of oxidative stress in baroreflex dysfunction is much more complex than a simple reflex dysfunction and needs to be further elucidated.

The two-kidney one-clip (2K1C) model of renovascular hypertensive is characterized by augmented angiotensin II, oxidative stress, inflammation, depressed baroreflex, sympathetic hyperactivity and endothelial dysfunction (Wang et al., 2005). The depressed arterial baroreflex sensitivity has also been reported in patients with renovascular hypertension (Gao et al., 2002).

For these reasons, it is reasonable to suggest that therapies or interventions with the potential to counteract the adverse effects of oxidative stress may be an important strategy for improving sympathetic baroreflex sensitivity and lower BP under hypertensive states (Guimaraes et al., 2012). In fact, recent studies from our research group have shown that an antioxidant therapy improves baroreflex sensitivity and BP control in hypertensive rats (Botelho-Ono et al., 2011; Guimaraes et al., 2012; Monteiro et al., 2012; Queiroz et al., 2012; Mendes-Junior et al., 2013). A recent meta-analysis suggested that baroreflex activation therapy could exert beneficial effects on BP in resistant hypertension. However, further experimental and clinical data are needed to confirm the application of baroreflex activation therapy under hypertensive conditions (Wallbach and Koziolek, 2017). Thus, we sought to identify antioxidant compounds that could be used as a potential anti-hypertensive strategy, mainly by modulating arterial baroreceptors.

Carboxymethyl-glucan (CM-G) is a water-soluble derivative of *Saccharomyces* spp. cell wall β(1-3)(1-6) glucan that appears to be translocated from the gastrointestinal (GI) tract into the systemic circulation by epithelial cells in the GI tract and their absorption kinetics differs among the different types of β-glucan (Vetvicka et al., 2015). Well-known by its antioxidant properties, CM-G reduces malondialdehyde levels in healthy men and it has an important role in the protection of biological membranes, probably by its main accepted mechanism that involves the capacity of CM-G to scavenge reactive oxygen species (Babincova et al., 2002; Araujo et al., 2015). Despite the free radical scavenging activity of CM-G, whether an oral treatment with CM-G could improve baroreflex sensitivity and ameliorate arterial hypertension in renovascular hypertensive rats remains unknown. Therefore, we assessed the ability of CM-G treatment to reduce arterial hypertension and restore baroreflex sensitivity in 2K1C hypertensive rats.

## Materials and Methods

### Animals

Thirty-four male Wistar rats (270–330 g) were used for the experiments, collectively housed in cages (3–4 rat/cage), maintained in a temperature-controlled room and subjected to a 12:12-hour light-dark cycle with free access to standard chow diet (Labina^®^, Purina, Paulinea, SP, Brazil) and water. All experimental procedures were approved by the Animal Care and Use Committee (CEUA, protocol #0604/14) of the Federal University of Paraiba, Brazil.

### General Experimental Protocol

**FIGURE 1:**
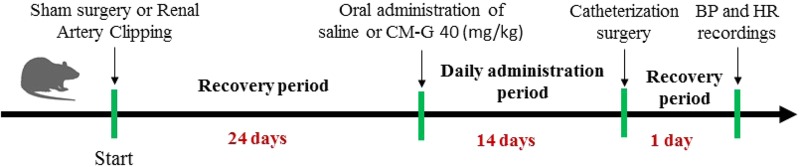
Experimental design performed in the study.

The rats were randomly assigned into four groups: i) Sham (submitted to sham surgery, *n* = 10); ii) 2K1C (subjected to renal artery clipping to induce renovascular hypertension, *n* = 10); iii) Sham + CM-G (submitted to sham surgery + CM-G treatment, *n* = 7); iv) 2K1C + CM-G (submitted to renal artery clipping to induce renovascular hypertension + CM-G treatment, *n* = 7). In the sham groups, saline was administered as placebo for 2 weeks. In the CM-G groups, carboxymethyl-glucan at a dose of 40 mg/kg was administered daily for 2 weeks. Saline and/or CM-G administration was performed by oral gavage. The treatments started 28 days after surgery and lasted for 2 weeks (**Figure 1**). After 2 weeks of treatment with saline or CM-G, baseline BP, heart rate (HR) records, baroreflex sensitivity, sympathetic vascular tone and spectral analysis of systolic arterial pressure were evaluated in each group.

### Renal Artery Clipping: Goldblatt (Two-Kidney, One Clip; 2K1C) Model Hypertension

In order to develop 2K1C renovascular hypertension, rats were anesthetized with ketamine and xylazine (75 and 10 mg⋅kg^-1^, i.p., respectively) and a midline abdominal incision was made. The right renal artery was exposed and isolated over a short segment by blunt dissection. A silver clip (0.20 mm internal diameter) was placed around the artery. The wound was closed and sutured. Sham-operated rats underwent a similar procedure but without permanent renal artery clipping, to serve as a control.

### Blood Pressure and Heart Rate Recordings

Six weeks after silver clip implantation in the renal artery or sham surgery, the rats were anesthetized with ketamine and xylazine (75 and 10 mg/kg, i.p., respectively) to insert polyethylene catheters in the femoral vein and artery for drug injection and arterial pressure recordings, respectively. BP and heart rate (HR) measurements were recorded 24h after catheter implantation in conscious rats using a pressure transducer coupled to an acquisition system (PowerLab; ADInstruments, CastleHill, NSW, Australia) connected to a computer running LabChart 5.0 software (ADInstruments).

### Baroreflex Sensitivity Test

After 50 min of BP and HR baseline recordings, baroreflex was evaluated using classical vasoactive drugs. Phenylephrine (8μg/Kg) and sodium nitroprusside (25μg/Kg) were given as an intravenous bolus injection to each group as previously reported (Braga et al., 2008; Guimaraes et al., 2012). Reflex changes in HR produced by vasoactive drug administration were quantified and plotted as changes in HR over changes in mean arterial pressure (ΔHR/ΔMAP). Data were analyzed by linear regression using Prism 6 (GraphPad Software, Inc., SanDiego, CA, United States); the slope of linear regression yield the baroreflex gain for each animal. In addition, during the baseline recordings without any intervention, spontaneous baroreflex sensitivity (SBRS) was calculated through the sequence method by computer software CardioSeries (v. 2.4).

### Evaluation of Sympathetic Tonus on the Vascular System

One hour after the baroreflex sensitivity test, the sympathetic vascular tone was evaluated by an intravenous injection of the ganglionic blocker hexamethonium (30 mg/kg, Sigma-Aldrich, São Paulo, SP, Brazil). The sympathetic tonus was calculated by the difference between the mean arterial pressure after the blockade and at the baseline.

### Power Spectral Analysis of Systolic Arterial Pressure Signals

A beat-by-beat time series of systolic arterial pressure (SAP) was extracted from baseline cardiovascular recordings (10 min epochs) of the pulsatile arterial pressure from rats in each group and the overall variability of those series was assessed using Fast Fourier Transform (FFT) spectral analysis (Cardioseries Software v2.4; www.danielpenteado.com). The spectra were integrated and the low-frequency component (LF, 0.2–0.75 Hz) was evaluated. LF from systolic arterial pressure is an index for sympathetic modulation.

### Determination of Kidneys and Heart Weight

Heart and kidneys were collected and weighed. Total organ mass (mg) was normalized by the body weight (g) giving an organ weight/body weight ratio index (ow/bw).

### Statistical Analysis

Results are expressed as mean ± SEM. Data were analyzed by *t*-test or one-way or two-way ANOVA followed by Tukey’s post hoc when appropriate. Statistical analyses were performed using Prism 6 (GraphPad Software^®^. Inc., La Jolla, CA, United States) and the differences were considered significant when *p* < 0.05.

## Results

### Body and Organs Weights

As shown in **Table 1**, the right clipped kidneys from both 2K1C groups presented a reduction in the kidney mass index when compared to the right kidneys from the sham-operated rats. In addition, the left non-clipped kidney mass index was increased in both 2K1C groups when compared to the left kidney from sham-operated rats as an expected compensatory functional effect. Absolute values for organ weights are also provided in **Table 1**.

**Table 1:** Absolute body weight (BW) and organ weight in sham rats or those treated with Carboxymethyl-glucan (CM-G).

Experimental groups	Initial BW (g)	Final BW (g)	Right kidney weight/BW (mg/g)	Left kidney weight/BW (mg/g)	Heart weight/BW (mg/g)	Right kidney weight (g)	Left kidney weight (g)
Sham + saline	169.1 ± 6.2	239.3 ± 5.1*	4.3 ± 0.14*	4.2 ± 0.13*	3.6 ± 0.24	1.0 ± 0.03	1.0 ± 0.04*
Sham + CM-G	194 ± 3.6	326.5 ± 8.8	3.8 ± 0.10	3.8 ± 0.1	3.8 ± 0.19	1.2 ± 0.06	1.2 ± 0.05
2K1C + saline	181.2 ± 5.2	272.5 ± 13.7	3.37 ± 0.22^#^	4.8 ± 0.17	4.3 ± 0.39	0.91 ± 0.07^#^	1.3 ± 0.05
2K1C + CM-G	184.8 ± 6.3	316.6 ± 9.8	2.6 ± 0.53^#^	4.5 ± 0.28	3.7 ± 0.10	0.83 ± 0.17^#^	1.4 ± 0.08

*^∗^ p < 0.05 vs. all other groups. ^#^p < 0.05 vs left side from the same group. Ratios were calculated by dividing the organ weight by the final body weight per animal. (Mean values with their standard errors)*

### CM-G Treatment Reduces Blood Pressure in 2K1C Rats

**FIGURE 2:**
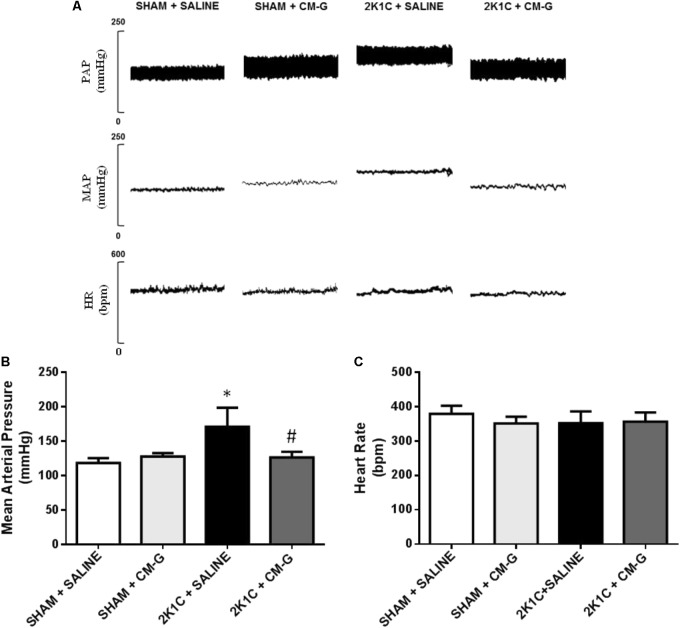
Oral administration of carboxymethyl-glucan (CM-G) reduces blood pressure in renovascular hypertensive rats **(A)** Original tracings from one representative animal from each group (Sham + Saline, Sham + CM-G, 2K1C + Saline, 2K1C + CM-G) showing pulsatile arterial pressure (PAP), mean arterial pressure (MAP) and heart rate (HR). **(B)** Effects of oral treatment for 2 weeks on MAP in 2K1C and sham rats. **(C)** Effects of oral treatment for 2 weeks on HR in 2K1C and sham rats. ^∗^ *P* < 0.05 2K1C + SALINE *vs* SHAM + SALINE; ^#^*P* < 0.05 2K1C + CM-G *vs* 2 K1C + SALINE.

**Figure 2A** shows original tracings of pulse arterial pressure (PAP), MAP and HR from one representative animal from each group. 2K1C rats exhibited high BP levels after six weeks of renal artery clipping in comparison to sham rats [171 ± 11 *vs.* 118 ± 4 mmHg, *p* < 0.05; (**Figure 2B**)]. Oral CM-G treatment for 2 weeks (40 mg/kg/day) in 2K1C rats effectively reduced MAP when compared to non-treated hypertensive rats [126 ± 4 *vs.* 171 ± 11 mmHg; *p* < 0.05; (**Figure 2B**)]. Regarding sham conditions, oral CM-G administration did not alter BP between groups. Lastly, HR was not different between groups (*p* > 0.05; **Figure 2C**).

### CM-G Treatment Restores Depressed Baroreflex Sensitivity in 2K1C Rats

**FIGURE 3:**
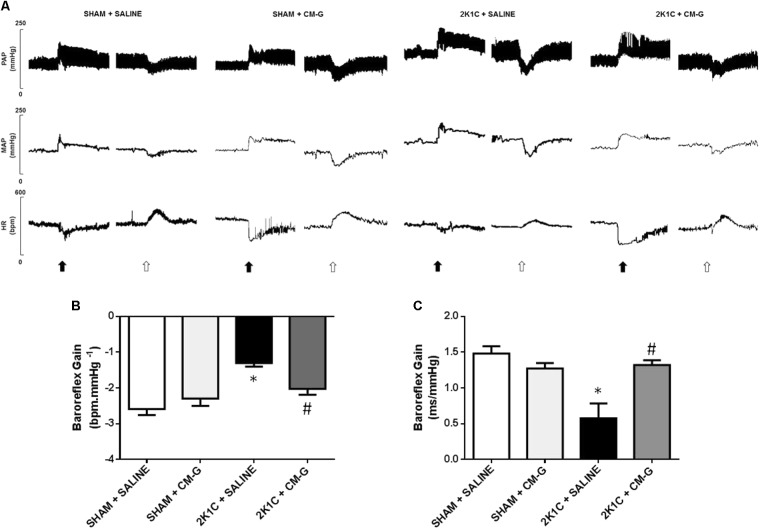
Oral administration of carboxymethyl-glucan (CM-G) restores baroreflex sensitivity in renovascular hypertensive rats **(A)** Original tracings from one representative animal from each group (Sham + Saline, Sham + CM-G, 2K1C + Saline, 2K1C + CM-G) showing changes in pulse arterial pressure (PAP), mean arterial pressure (MAP) and heart rate (HR) in response to phenylephrine (8 μg/Kg, i.v., black arrows) and sodium nitroprusside (25 μg/Kg, i.v., open arrows). **(B)** Effects of oral administration of CM-G for 2 weeks on pharmacologically evoked baroreflex sensitivity. **(C)** Effects of oral administration of CM-G for 2 weeks on spontaneous baroreflex sensitivity. ^∗^ *P* < 0.05 2K1C + SALINE *vs* SHAM + SALINE; ^#^*P* < 0.05 2K1C + CM-G *vs* 2 K1C + SALINE

Original tracings from one representative animal from each group showing the changes in BP and HR in response to administration of vasoactive drugs are illustrated in **Figure 3A**. 2K1C hypertensive rats presented a reduction in baroreflex gain when compared to the sham group (-1.30 ± 0.10 *vs.* -2.59 ± 0.17 bpm.mmHg^-1^, *p* < 0.05; **Figures 3B,C**). Daily CM-G treatment in 2K1C hypertensive rats restored the depressed baroreflex sensitivity when compared to non-treated hypertensive rats [-2.03 ± 0.16 *vs.* -1.30 ± 0.10 bpm.mmHg^-1^; *p* < 0.05; (**Figures 3B,C**)]. Similar to baseline BP results, the CM-G treatment in sham rats did not alter baroreflex sensitivity (*p* > 0.05, **Figures 3B,C**). Regarding the spontaneous baroreflex sensitivity (SBRS), 2K1C rats exhibited reduced SBRS when compared to sham rats (0.58 ± 0.08 *vs.* 1.48 ± 0.04 ms.mmHg^-1^, *p* < 0.05) and oral CM-G treatment improved the SBRS in 2K1C rats when compared to non-treated hypertensive rats (1.32 ± 0.03 vs. 0.58 ± 0.08 ms.mmHg^-1^; *p* < 0.05). No changes in SBRS were observed in the sham rats.

### CM-G Treatment Reduces Sympathetic Tone in 2K1C Rats

**FIGURE 4:**
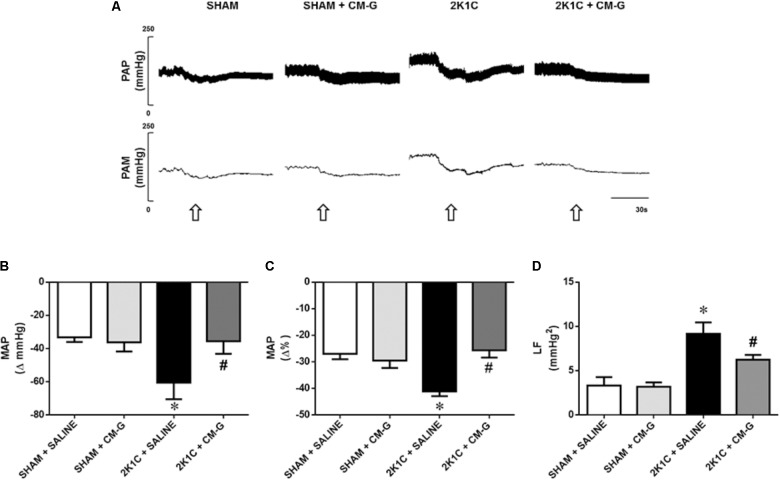
Oral administration of carboxymethyl-glucan (CM-G) reduces sympathetic tone in renovascular hypertensive rats **(A)** Original tracings from one representative animal from each group (Sham + Saline, Sham + CM-G, 2K1C + Saline, 2K1C + CM-G) showing changes in pulse arterial pressure (PAP) and mean arterial pressure (MAP) in response to ganglionic blocker with hexamethonium (30 μg/Kg, i.v., open arrows). **(B)** Effects of oral administration of CM-G for 2 weeks on the evaluation of delta change of the MAP after blockade with hexamethonium. **(C)** Data now presented as percentage changes in MAP after blockade with hexamethonium. (d) Values of low frequency component (LF) of each group showing that oral administration of carboxymethyl-glucan (CM-G) reduces neurogenic sympathetic vasomotor activity in renovascular hypertensive rats. ^∗^ *P* < 0.05 2K1C + SALINE *vs* SHAM + SALINE; ^#^*P* < 0.05 2K1C + CM-G *vs* 2 K1C + SALINE.

Representative tracings of PAP and MAP after hexamethonium application are shown in **Figure 4A**. After ganglionic blockage, the 2K1C hypertensive rats showed a greater fall in blood pressure [ΔMAP, -60 ± 5 vs. -33 ± 2 mmHg, *p* < 0.05; (**Figure 4B**)] when compared to the sham rats. Oral CM-G treatment in 2K1C rats for 2 weeks attenuated the fall in blood pressure when compared to non-treated 2K1C rats [-35 ± 3 vs. -60 ± 5 mmHg; *p* < 0.05; (**Figure 4B**)], suggesting a reduction in sympathetic tone. The percentage changes in blood pressure among groups after hexamethonium is illustrated in **Figure 4C**. CM-G treatment had no effect on the sympathetic tone in sham rats when compared to non-treated sham rats.

### CM-G Treatment Reduces Neurogenic Sympathetic Vasomotor Activity in 2K1C Rats

Renovascular hypertensive rats exhibited an increase in the magnitude of oscillatory components in the low-frequency (LF) range of SAP when compared to the sham group [9.16 ± 0.52 vs. 3.32 ± 0.38 mmHg^2^, *p* < 0.05; (**Figure 4D**)]. Oral treatment with CM-G for 2 weeks reduced LF bands in 2K1C rats in comparison to non-treated renovascular hypertensive rats [6.23 ± 0.46 vs. 9.16 ± 0.52 mmHg^2^; *p* < 0.05, (**Figure 4D**)].

## Discussion

We have demonstrated that oral CM-G treatment for 2 weeks reduced arterial hypertension and restored depressed baroreflex sensitivity in conscious renovascular hypertensive rats. These benefits seem to be related to a reduction in sympathetic tone.

The pre-clinical and clinical use of CM-G has been proposed as part of a combination therapy for a variety of diseases mainly because of its strong capability to scavenge reactive oxygen species (ROS) (Magnani et al., 2011; Araujo et al., 2015). For example, it has been reported that oral administration of CM-G helps to protect DNA against damage in prostate cancer patients (Magnani et al., 2011) and reduced the malondialdehyde serum levels in healthy individuals (Araujo et al., 2015). In addition, it has been reported that the daily oral treatment with CM-G (20 mg/kg) for eight days reduced interleukin 8 (IL-8), improved vascular response to nitric oxide and exhibited anti-aggregation activity. This suggests that CM-G could have a beneficial effect on the vascular system (Bezerra et al., 2017).

Considering the antioxidant capacity of the CM-G, we may suggest that our treatment with CM-G might have reduced BP in the 2K1C rats, secondary to the capability of scavenging reactive oxygen species. In agreement with other studies that used acute antioxidant treatment for reducing BP in hypertensive rats (Costa et al., 2009; Guimaraes et al., 2012; Mengal et al., 2016), our study showed that CM-G treatment may be a relevant strategy to ameliorate BP in renovascular hypertension. However, we point out that CM-G treatment reduced blood pressure after 2 weeks of treatment, while most studies using antioxidant intervention with similar dose found reduction in BP only after 3 or 4 weeks of treatment (Alves et al., 2015; de Queiroz et al., 2015; Mengal et al., 2016). A recent study examined the antioxidant effects of green tea demonstrated that 1-week administration of the tea reduced blood pressure and sympathoexcitation in hypertensive rats (Garcia et al., 2017). Similarly, daily treatment with quercetin (25 mg/kg, for 1 week) reduced blood pressure in spontaneously hypertensive rats (Monteiro et al., 2012).

One of the key mechanisms in controlling blood pressure in health and disease is the baroreflex (Grassi et al., 1998). Baroreceptors located in the carotid sinuses and aortic arch detect changes in blood pressure and trigger reflex autonomic adjustments that buffer alterations in blood pressure (Malpas et al., 1997; Kanbar et al., 2007; Salgado et al., 2007). In pathological conditions such as hypertension, there is impairment in the autonomic control of blood pressure resulting in changes in baroreflex sensitivity (Gao et al., 2002; Nagai et al., 2003; Martinka et al., 2005).

The 2K1C model increased levels in ANG-II circulation, which might act in specific areas of the brainstem, augmenting oxidative stress and promoting autonomic dysfunction and reduction of baroreflex sensitivity (Heitzer et al., 1999). Our results showed that CM-G treatment improved the baroreflex sensitivity in animals with renovascular hypertension. Considering the well-known efficacy of antioxidant therapies for improving of baroreflex sensitivity in ANG-II-dependent hypertension models (Botelho-Ono et al., 2011; Queiroz et al., 2012; Mendes-Junior et al., 2013; Alves et al., 2015), it is probable that the improvement of baroreflex sensitivity was a result of the antioxidant therapy promoted by the oral CM-G treatment. In fact, previous studies have demonstrated that the antioxidant therapy had no effect on baroreflex function in normotensive animals (Li et al., 1996; Pickering et al., 2008), but improved the baroreflex in hypertensive rats (Guimaraes et al., 2012; Monteiro et al., 2012; Mendes-Junior et al., 2013). This suggests that antioxidant administration in the absence of oxidative stress has no influence on baroreflex sensitivity.

Additionally, CM-G treatment plays an important role in reducing sympathetic tone in rats with renovascular hypertension. Reactive oxygen species (ROS) in the brainstem have a potential role in the modulation of sympathetic activity and BP in hypertensive rats, suggesting that oxidative stress can contribute to higher sympathetic activity and hypertension (Malpas, 2010; Chan and Chan, 2014). Given this, further study is needed to see whether the reduced sympathetic overactivity was a consequence of the re-establishment of baroreflex sensitivity or if CM-G treatment could act as antioxidant therapy directly on the neuronal network involved in sympathetic control (Huber and Schreihofer, 2010). One possible limitation of our study is that the sympathetic modulation of blood pressure was evaluated one hour after the vasoactive drugs protocol. One could argue that using vasoactive drugs to evaluate baroreflex could trigger vasopressin release. However, it is important to note that vasopressin release is far more sensitive to changes in plasma osmolarity than to changes in blood volume. Therefore, repeated doses of the vasoactive drugs would be needed to trigger effective changes in vasopressin release, which was not the case. The use of a single bolus injection of vasoactive drugs in very small volumes/concentrations for the baroreflex sensitivity test allows a considerable margin of safety regarding the intervention of other mechanisms such as osmolality changes and release of vasopressin.

In order to further assess the idea that CM-G treatment reduces arterial hypertension, we performed the spectral analysis of systolic blood pressure. Our findings indicated that CM-G treatment significantly reduces the LF component of the spectral analysis. The LF component is a well-accepted index of sympathetic modulation (Inoue et al., 1991). Therefore, although we were not able to perform direct recordings of the sympathetic activity, which would be the gold standard for sympathetic evaluation, both the hexamethonium and the spectral analysis data suggest that CM-G treatment reduces sympathetic activity in renovascular hypertensive rats.

Further studies investigating the underlying mechanisms involved in CM-G treatment and reduction of arterial hypertension, improvement of the baroreflex sensitivity and the involvement of the sympathetic tone will be needed. Our experience leads to the following suggestions. First, it is important knowing if CM-G alters antioxidant enzymes, such as superoxide dismutase, catalase, glutathione peroxidase and glutathione reductase activities, in the vascular or brainstem level. Second, we need to understand if CM-G can improve other control mechanisms of arterial blood pressure, such as the peripheral chemoreflex or renin-angiotensin-aldosterone system.

In summary, we reported that acute oral CM-G treatment reduced arterial hypertension and restored baroreflex sensitivity via reduction of the sympathetic tone in renovascular hypertensive rats. In fact, the precise site of action where CM-G therapy produces its beneficial effects in order to ameliorate hypertension remains unknown. However, we have identified one more antioxidant compound with baroreceptor modulation properties that could be used in the future as an additional therapy for renovascular hypertension.

## Author Contributions

AC-G and VB designed the experiments. AC-G and DG performed and analyzed the experiments. AC-G, DG, JB, and VB wrote the manuscript. AC-G, DG, JB, BK, RC-G, JC, MM, and VB reviewed the manuscript.

## Conflict of Interest Statement

The authors declare that the research was conducted in the absence of any commercial or financial relationships that could be construed as a potential conflict of interest.

## Abbreviations

ANG II: Angiotensin II
BRS: Baroreflex sensitivity
BP: Blood pressure
CM-G: Carboxymethyl-glucan
HR: Heart rate
LF: Low-frequency
MAP: Mean arterial pressure
PAP: Pulsatile arterial pressure
ROS: Reactive oxygen species
SBRS: Spontaneous baroreflex sensitivity
SAP: Systolic arterial pressure
2K1C: Two-kidney one-clip

## References

[B1] Alves N. F. Porpino S. K. Monteiro M. M. Gomes E. R. Braga V. A. (2015). Coconut oil supplementation and physical exercise improves baroreflex sensitivity and oxidative stress in hypertensive rats. 40 393–400. 10.1139/apnm-2014-035 25659569

[B2] Araujo V. B. de Melo A. N. de Souza N. T. da Silva V. M. Castro-Gomez R. H. Silva A. S. (2015). Oral intake of Carboxymethyl-Glucan (CM-G) from yeast (*Saccharomyces uvarum*) reduces malondialdehyde levels in healthy men. 20 14950–14958. 10.3390/molecules200814950 26287149 PMC6332209

[B3] Babincova M. Bacova Z. Machova E. Kogan G. (2002). Antioxidant properties of carboxymethyl glucan: comparative analysis. 5 79–83. 10.1089/109662002760178159 12487754

[B4] Bezerra L. S. Magnani M. Castro-Gomez R. J. H. Cavalcante H. C. Silva T. Vieira R. L. P. (2017). Modulation of vascular function and anti-aggregation effect induced by (1––>3) (1––>6)-beta-d-glucan of Saccharomyces cerevisiae and its carboxymethylated derivative in rats. 69 448–455. 10.1016/j.pharep.2017.01.002 28319748

[B5] Botelho-Ono M. S. Pina H. V. Sousa K. H. Nunes F. C. Medeiros I. A. Braga V. A. (2011). Acute superoxide scavenging restores depressed baroreflex sensitivity in renovascular hypertensive rats. 159 38–44. 10.1016/j.autneu.2010.07.025 20719579

[B6] Braga V. A. Burmeister M. A. Sharma R. V. Davisson R. L. (2008). Cardiovascular responses to peripheral chemoreflex activation and comparison of different methods to evaluate baroreflex gain in conscious mice using telemetry. 295 R1168–R1174. 10.1152/ajpregu.90375.2008 18667715 PMC2576088

[B7] Cavalcanti C. O. Alves R. R. de Oliveira A. L. Cruz J. C. de Franca-Silva M. S. Braga V. A. (2016). Inhibition of PDE5 restores depressed baroreflex sensitivity in renovascular hypertensive rats. 28:15. 10.3389/fphys.2016.00015 26858657 PMC4729906

[B8] Chan S. H. Chan J. Y. (2014). Brain stem NOS and ROS in neural mechanisms of hypertension. 20 146–163. 10.1089/ars.2013.5230 23418728

[B9] Costa C. A. Amaral T. A. Carvalho L. C. Ognibene D. T. da Silva A. F. Moss M. B. (2009). Antioxidant treatment with tempol and apocynin prevents endothelial dysfunction and development of renovascular hypertension. 22 1242–1249. 10.1038/ajh.2009.186 19779472

[B10] de Queiroz T. M. Xia H. Filipeanu C. M. Braga V. A. Lazartigues E. (2015). alpha-Lipoic acid reduces neurogenic hypertension by blunting oxidative stress-mediated increase in ADAM17. 309 H926–H934. 10.1152/ajpheart.00259.2015 26254330 PMC4591409

[B11] Gao S. A. Johansson M. Rundqvist B. Lambert G. Jensen G. Friberg P. (2002). Reduced spontaneous baroreceptor sensitivity in patients with renovascular hypertension. 20 111–116. 10.1097/00004872-200201000-00016 11791033

[B12] Garcia M. L. Pontes R. B. Nishi E. E. Ibuki F. K. Oliveira V. Sawaya A. C. (2017). The antioxidant effects of green tea reduces blood pressure and sympathoexcitation in an experimental model of hypertension. 35 348–354. 10.1097/HJH.0000000000001149 28005704

[B13] Grassi G. Cattaneo B. M. Seravalle G. Lanfranchi A. Mancia G. (1998). Baroreflex control of sympathetic nerve activity in essential and secondary hypertension. 31 68–72. 10.1161/01.HYP.31.1.68 9449393

[B14] Guimaraes D. D. Carvalho C. C. Braga V. A. (2012). Scavenging of NADPH oxidase-derived superoxide anions improves depressed baroreflex sensitivity in spontaneously hypertensive rats. 39 373–378. 10.1111/j.1440-1681.2012.05679.x 22283703

[B15] Heitzer T. Wenzel U. Hink U. Krollner D. Skatchkov M. Stahl R. A. (1999). Increased NAD(P)H oxidase-mediated superoxide production in renovascular hypertension: evidence for an involvement of protein kinase C. 55 252–260. 10.1046/j.1523-1755.1999.00229.x 9893134

[B16] Huber D. A. Schreihofer A. M. (2010). Attenuated baroreflex control of sympathetic nerve activity in obese Zucker rats by central mechanisms. 588(Pt 9), 1515–1525. 10.1113/jphysiol.2009.186387 20211978 PMC2876806

[B17] Inoue K. Miyake S. Kumashiro M. Ogata H. Ueta T. Akatsu T. (1991). Power spectral analysis of blood pressure variability in traumatic quadriplegic humans. 260 H842–H847. 10.1152/ajpheart.1991.260.3.H842 2000979

[B18] Julien C. (2008). Baroreflex control of sympathetic nerve activity and blood pressure variability. 35 512–515. 10.1111/j.1440-1681.2008.04907.x 18307752

[B19] Kanbar R. Orea V. Barres C. Julien C. (2007). Baroreflex control of renal sympathetic nerve activity during air-jet stress in rats. 292 R362–R367. 10.1152/ajpregu.00413.2006 16973933

[B20] Li Z. Mao H. Z. Abboud F. M. Chapleau M. W. (1996). Oxygen-derived free radicals contribute to baroreceptor dysfunction in atherosclerotic rabbits. 79 802–811. 10.1161/01.RES.79.4.802 8831504

[B21] Magnani M. Castro-Gomez R. J. Mori M. P. Kuasne H. Gregorio E. P. Libos F. (2011). Protective effect of carboxymethyl-glucan (CM-G) against DNA damage in patients with advanced prostate cancer. 34 131–135. 10.1590/S1415-47572010005000103 21637556 PMC3085359

[B22] Malpas S. C. (2010). Sympathetic nervous system overactivity and its role in the development of cardiovascular disease. 90 513–557. 10.1152/physrev.00007.2009 20393193

[B23] Malpas S. C. Groom A. S. Head G. A. (1997). Baroreflex control of heart rate and cardiac hypertrophy in angiotensin II-induced hypertension in rabbits. 29 1284–1290. 10.1161/01.HYP.29.6.1284 9180630

[B24] Martinka P. Fielitz J. Patzak A. Regitz-Zagrosek V. Persson P. B. Stauss H. M. (2005). Mechanisms of blood pressure variability-induced cardiac hypertrophy and dysfunction in mice with impaired baroreflex. 288 R767–R776. 10.1152/ajpregu.00445.2004 15563577

[B25] Mendes-Junior L. Monteiro M. M. Carvalho A. S. de Queiroz T. M. Braga Vde A. (2013). Oral supplementation with the rutin improves cardiovagal baroreflex sensitivity and vascular reactivity in hypertensive rats. 38 1099–1106. 10.1139/apnm-2013-0091 24053516

[B26] Mengal V. Silva P. H. Tiradentes R. V. Santuzzi C. H. de Almeida S. A. Sena G. C. (2016). Aliskiren and l-arginine treatments restore depressed baroreflex sensitivity and decrease oxidative stress in renovascular hypertension rats. 39 769–776. 10.1038/hr.2016.61 27383506

[B27] Monteiro M. M. Franca-Silva M. S. Alves N. F. Porpino S. K. Braga V. A. (2012). Quercetin improves baroreflex sensitivity in spontaneously hypertensive rats. 17 12997–13008. 10.3390/molecules171112997 23117438 PMC6269113

[B28] Nagai R. Nagata S. Fukuya F. Higaki J. Rakugi H. Ogihara T. (2003). Changes in autonomic activity and baroreflex sensitivity with the hypertension process and age in rats. 30 419–425. 10.1046/j.1440-1681.2003.03852.x 12859436

[B29] Pickering A. E. Simms A. E. Paton J. F. (2008). Dominant role of aortic baroreceptors in the cardiac baroreflex of the rat in situ. 142 32–39. 10.1016/j.autneu.2008.03.009 18479978

[B30] Queiroz T. M. Guimaraes D. D. Mendes-Junior L. G. Braga V. A. (2012). alpha-lipoic acid reduces hypertension and increases baroreflex sensitivity in renovascular hypertensive rats. 17 13357–13367. 10.3390/molecules171113357 23143148 PMC6268197

[B31] Salgado H. C. Barale A. R. Castania J. A. Machado B. H. Chapleau M. W. Fazan R. (2007). Baroreflex responses to electrical stimulation of aortic depressor nerve in conscious SHR. 292 H593–H600. 10.1152/ajpheart.00181.2006 16951050

[B32] Vetvicka V. Vannucci L. Sima P. (2015). Role of β-glucan in biology of gastrointestinal tract. 1:e129.

[B33] Wallbach M. Koziolek M. J. (2017). Baroreceptors in the carotid and hypertension-systematic review and meta-analysis of the effects of baroreflex activation therapy on blood pressure. 10.1093/ndt/gfx279 [Epub ahead of print]. 29136223

[B34] Wang D. S. Xie H. H. Shen F. M. Cai G. J. Su D. F. (2005). Blood pressure variability, cardiac baroreflex sensitivity and organ damage in experimentally hypertensive rats. 32 545–552. 10.1111/j.1440-1681.2005.04229.x 16026514

